# Editorial: Impact of apathy on aging and age-related neuropsychiatric disorders

**DOI:** 10.3389/fpsyt.2023.1341182

**Published:** 2023-12-13

**Authors:** Amer M. Burhan

**Affiliations:** ^1^Ontario Shores Centre for Mental Health Sciences, Whitby, ON, Canada; ^2^Department of Psychiatry, Temerty Faculty of Medicine, University of Toronto, Toronto, ON, Canada

**Keywords:** apathy, neurocognitive disorder, neuropsychiatric symptoms, transcranial magnetic stimulation (TMS), neuroimaging, scales (or assessment), stroke, Parkinson

The global population is aging with those over the age of 60 doubling between the years 2015 and 2050 (1). Apathy is a prevalent, persistent, and difficult-to-manage symptom of many brain disorders that are common in old age. Diagnostic criteria for apathy in brain disorders were revised and published by “The 2018 International Consensus Group” (2). The criteria include a quantitative reduction of goal-directed activity across behavioral, cognitive, emotional, or social domains from the patient's baseline that has been there most of the time over the last 4 weeks, that causes significant impairment and is not exclusively explained by other factors like physical disability, the physiological effect of substances, and environmental factors. There is ongoing work to better profile apathy in the context of dementia [i.e., neurocognitive disorders] (3), stroke (4), Parkinson's disease (5), and TBI (6).

In this Research Topic focusing on the impact of apathy in older adults with neuropsychiatric disorders, we invited colleagues from around the world to share their perspectives on this under-studied and under-reported syndrome. We got contributions from several countries around the world and in different areas of work. Manera et al. from Université Côte d'Azur, CobTeK, Nice, France, published an application to assess apathy as a novel alternative to the clinical rating scale and showed that in their cohort of 227 older adults with mild and major neurocognitive disorders, the “interest game” has the ability to detect significant apathy with a sensitivity and specificity of 0.68 and 0.65, respectively. Innovative ways to assess apathy are critically needed to get to the core symptom of diminished interest and work of this sort is an important step in this direction. Yan et al. from several universities in China conducted a thorough meta-analysis of structural neuroimaging studies in apathy across healthy and brain disorders including neurodegenerative illnesses and traumatic brain injury. They identified several brain areas that are likely involved in the mechanism of apathy. This work will likely pave the way for more work to further elucidate the basic brain mechanism of apathy and potential therapeutic targets for interventions including therapeutic brain stimulation. Indeed, the paper by Espiritu et al., with contributions from the Philippines, Japan, and Canada, presented a systematic review of the potential therapeutic benefit of repetitive transcranial magnetic stimulation in apathy across different brain disorders that are common in old age. The paper reports limited evidence but potential benefits of rTMS in Alzheimer's disease, primary progressive aphasia, mild cognitive impairment, and chronic stroke. More work is being done to confirm the role of brain stimulation for apathy in neurocognitive and other brain disorders and hopefully will be reported in the near future. While there was no contribution to this special edition on the role of pharmacological interventions in apathy, some work has been published elsewhere on that topic and showed promise from several pharmacological agents as monotherapy or in combination like acetylcholine esterase inhibitors and methylphenidate, a stimulant (7).

Apathy is a final common pathway of different pathologies that affect the cognitive-motivational networks in the brain. Like many illness manifestations, apathy is the product of the abnormal balance between cognitive-motivational resources, and demands of internal and external factors. Cognitive-motivational network involvement has been found to be the common underlying mechanism of many brain disorders manifesting as apathy (8). Like any other illness manifestation, it is not “all or none” but rather a spectrum of severity that is considered “illness” when the level of impairment significantly impacts the quality of life and/or function. It is important, on the other hand, to consider processes that contribute to this impairment. These factors can be external factors, like lack of opportunity to participate due to physical disability or limited support, or internal factors like competing brain network activation such as negative emotional or pain networks. The paper by Zhong et al. from Chengdu University in China while didn't specifically focus on apathy, it highlighted the link between depression, which is commonly co-morbid with apathy, and sarcopenia, the hallmark of frailty in older adults. The authors utilized large cohort data and performed a Mendelian randomization methodology demonstrating a causal connection between depression and sarcopenia. This work contributes to our understanding of genetic and environmental factors that can affect the ability of older adults to engage in activities and is essential to consider when assessing and providing holistic treatment to older adults.

Figure 1 outlines a model of what could be operating in the process from wanting to do something new, plan/prepare, initiate, persist in doing, getting a reward, and then wanting to do it again due to the positive reinforcement from the rewarding experience.

**Figure 1:**
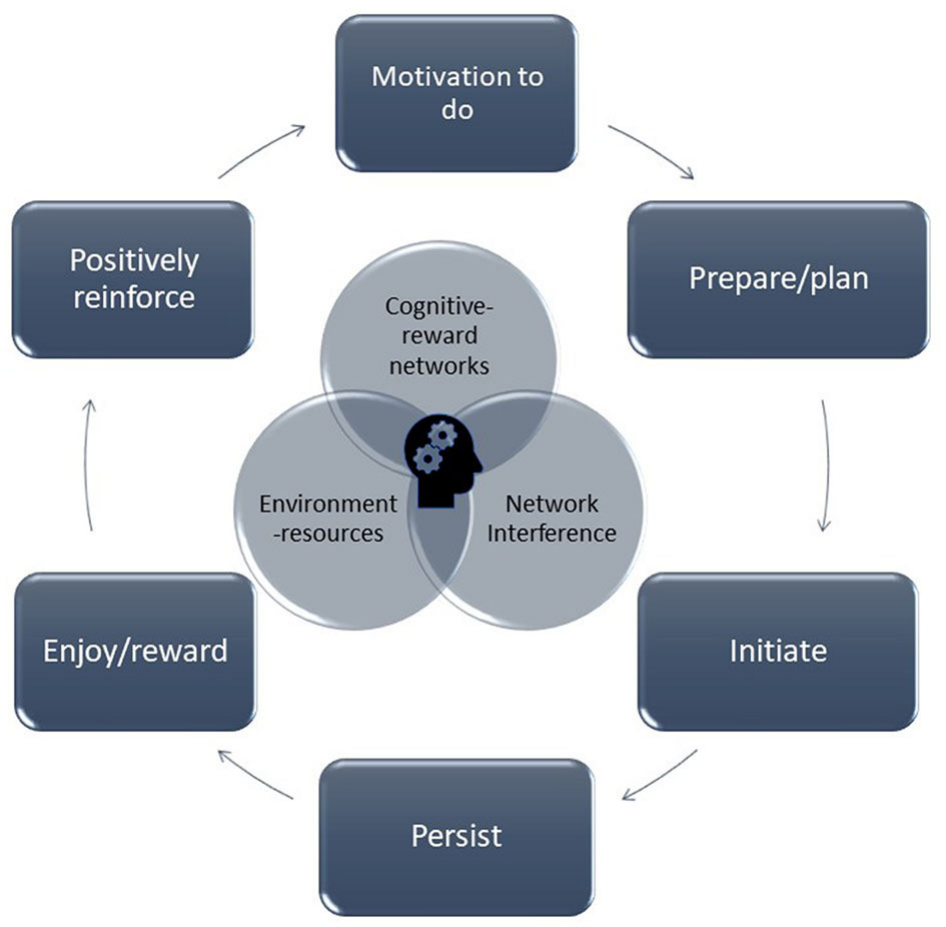
The cycle of motivation-reinforcement is depicted whereby the motivation to start an activity leads to preparation and planning, initiation, persistent in the activity, feel the reward, which ultimately result in positive reinforcement to do the activity or other activities again.

This Research Topic issue is a step toward understanding the impact of apathy on the aging population worldwide, much more work is needed and we look forward to seeing more being published on the results of current and future studies including studies that improve measurements and leverage technology and data modeling (including deep learning and consumer devices), better define therapeutic targets based on better understanding of underlying brain mechanisms, and combine different interventions to address the complexity of factors that contribute to this syndrome.

## Author contributions

AB: Conceptualization, Writing – original draft, Writing – review & editing.
